# Occurrence of Orofacial and Dental Injuries in Rugby: Systematic Review and Meta‐Analysis

**DOI:** 10.1002/cre2.70315

**Published:** 2026-03-23

**Authors:** Marc Chaboussie, Alexandre Baudet

**Affiliations:** ^1^ Université de Lorraine, faculté d'odontologie Vandœuvre‐lès‐Nancy France; ^2^ CHRU‐Nancy, service d'odontologie Nancy France

**Keywords:** dental, injury, meta‐analysis, orofacial, rugby, systematic review, trauma

## Abstract

**Objectives:**

The main aim of this systematic review and meta‐analysis was to report the prevalences of orofacial and dental injuries among rugby players and to discuss the prevention of these injuries through mouthguard use.

**Methods:**

A literature search of the PubMed, Scopus, EMBASE, Cochrane Library and Dentistry & Oral Science Sources (DOSS) databases was performed to identify eligible studies from 1998 to 31^st^ March 2025. This review was conducted on studies reporting the prevalence or incidence of orofacial or dental injuries among rugby players.

**Results:**

In total, 268 records were screened for eligibility, and 16 studies met the inclusion criteria. The overall prevalence of orofacial injuries was 40.4% (95% CI: 38.5–42.2) with the higher prevalence (70.2%) in a study of 2010 among male rugby players of 17–18 years. The most common orofacial injuries involve the soft tissues. The overall prevalence of dental injuries was 19.6% (95% CI: 18.1–21.3). A fractured tooth was the most common dental injury. Mouthguards were mainly worn during competition, less during training. The certainty in cumulative evidence was considered to be very low.

**Conclusion:**

This review highlights a high rate of orofacial and dental injuries in rugby. It is necessary to increase awareness and the use of protective mouthguards to enhance prevention.

## 1 Introduction

Rugby is a contact sport practiced by many players. According to World Rugby, participation in rugby increased by 11% worldwide in 2023, reaching 8.5 million players from 132 member countries. Rugby benefited from the success of the last Rugby World Cup in 2023 with record ticket sales to attend the matches, television audiences and following official accounts on social networks (Rugby World Cup 2025).

However, this sport is not without risk, as rugby exposes participants to numerous injuries, even in young players. A meta‐analysis on the incidence of concussions in youth sports placed rugby among the sports with a high incidence rate of concussions (Pfister et al. 2016). It was estimated that rugby players have a 10% chance of injury during a playing season and a 50% chance of injury during their playing career (Singh Dhillon et al. 2014). A meta‐analysis on the incidence of injuries among men playing amateur rugby showed that injuries can occur at different locations on the body and that the incidence of injuries can vary depending on the position of the player on the field and on the level of the player (Yeomans et al. 2018). Regarding professional rugby unions, a meta‐analysis of elite men's rugby showed that the estimated mean number of days missed per match injury was 27 days (Williams et al. 2022).

Rugby players are especially exposed to injuries affecting the orofacial and dental regions. During matches, the head was the most common injury location (16.7%) (Williams et al. 2022). Orofacial and dental trauma are major concerns due to the physical nature and direct contact inherent to rugby. These injuries can have long‐term consequences, affecting the oral health of players and leading to considerable costs in terms of medical care and rehabilitation, as well as insurance. However, the proportion of dental injuries in rugby unions declined in the early 21st century. Indeed, since 1998, it has become compulsory for rugby players of all levels to wear mouthguards during matches (Welch et al. 2010).

A comprehensive and systematic understanding of the occurrence of orofacial and dental injuries in rugby practice is lacking in the literature. This systematic review aimed to analyze in depth existing studies on the occurrence of orofacial and dental injuries in rugby. The second aim was to describe the use of mouthguards, and the knowledge needed to manage a tooth injury in rugby.

## 2 Materials and Methods

Relevant studies were reviewed based on the Preferred Reporting Items for Systematic reviews and Meta‐Analyses (PRISMA) statement (Page et al. 2021).

### 2.1 Data Sources and Search Strategy

Systematic literature searches were conducted in five databases: PubMed, Scopus, EMBASE, the Cochrane Library, and Dentistry & Oral Science Sources (DOSS). The last database search took place on 31^st^ March 2025 and included all relevant publications from 1998 (year when it became compulsory for rugby players to wear mouthguards during matches) to this date.

The first search of these databases was conducted using the following search equation: “rugby” AND (“dental” OR “orofacial”) AND (“trauma” OR “injury”). In addition, the bibliographies of the included studies and previous reviews were searched to identify other potentially eligible articles.

The keywords used come from HeTOP (Health Terminology/Ontology Portal) and belong to the Medical Subject Heading (MeSH) terminology, a reference thesaurus in the biomedical field.

### 2.2 Selection Process and Eligibility Criteria

The selection criteria for the review were decided upon by the two authors. The authors independently evaluated each title, abstract and full article against the selection criteria (Supporting Information S1). Subsequently, they compared and shared their selection and mediated together in the case of any disagreement.

After removal of duplicates, titles and abstracts were screened for eligibility. Studies were limited to observational epidemiological studies (retrospective, longitudinal, cohort, ecological, and cross‐sectional studies) in English‐language articles on orofacial trauma and/or dental injuries during rugby. Thus, books, review articles, qualitative studies, case reports and case series were excluded.

Following title and abstract screening, the full texts of all potentially eligible articles were retrieved to determine if they met the inclusion/exclusion criteria. Studies reporting the prevalence or incidence of orofacial or dental injuries among rugby players were included. Individuals of all genders of any age and all playing levels were eligible for inclusion.

### 2.3 Data Collection Process and Data Items

The two authors extracted data from the eligible articles and recorded them in Microsoft Excel. The following data were recorded: author, year of publication, title, country, year/period of study, study design, sample characteristics, sample size, method of data collection, outcomes, prevalence and rate of orofacial injuries, prevalence of dental injuries, prevalence of injuries according to experience and according to mouthguard use, prevalence of mouthguard use, relative risk, mouthguard form, time to wear a mouthguard, complications, management of tooth injury, and conclusion.

The primary outcome measure was the prevalence of dental and orofacial injuries. The secondary outcome measure included the prevalence of mouthguard use among rugby players.

### 2.4 Grading of Recommendations Assessment, Development and Evaluation (GRADE)

The certainty in cumulative evidence available was assessed according to the Grading of Recommendations Assessment, Development and Evaluation (GRADE) criteria (Guyatt et al. 2008). An evidence profile was produced by using the online software GRADEpro (McMaster University).

### 2.5 Risk of Bias Assessment

Quality assessment was carried out by the two authors independently by applying the critical appraisal checklist proposed by the Joanna Briggs Institute (JBI) (Barker et al. 2023). The use of the “JBI checklist” in this systematic review is justified by its reputation as a robust methodological tool specially designed to assess the quality of the included studies. The JBI checklist provides a systematic approach to examining risks of bias in the design and conduct of studies, thereby contributing to a comprehensive assessment of the validity of results. The JBI offers a checklist for each type of study to be analyzed. For this review, we chose to use a single checklist, the cross‐sectional studies checklist, which is most appropriate for the majority of studies in our literature review. This approach simplified the presentation and comparability of different studies. It comprised eight questions, and each item was documented as yes, no, unclear, or not applicable. Studies were characterized as follows: low risk of bias, if more than 70% of the scores were “yes”; moderate risk of bias, if “yes” scores were between 50% and 69%; and high risk of bias, if “yes” scores were less than 49%.

### 2.6 Statistical Analysis

By Cohen's Kappa, it was observed that there was a moderate agreement between the two authors regarding the screening of titles and abstracts (*κ* = 0.71) and a strong agreement regarding the risk of bias assessment (*κ* = 0.86).

The meta‐analysis was conducted using R version 2025.05.1+513 with the R package “meta.” The function “metaprop” was used to obtain the overall prevalences of orofacial and dental injuries from the studies. The function built a generalized linear mixed‐effects model which fitted a logistic regression model including random‐effects model. The 95% confidence intervals (95% CIs) for individual studies were based on a t‐distribution and the Hartung–Knapp adjustment was used. Heterogeneity was evaluated using Cochrane Q statistics and the τ^2^ and I^2^ indices. Statistical significance was set at *p* < 0.05. Forest plots were constructed using the function “meta::forest.”

## 3 Results

**Figure 1:**
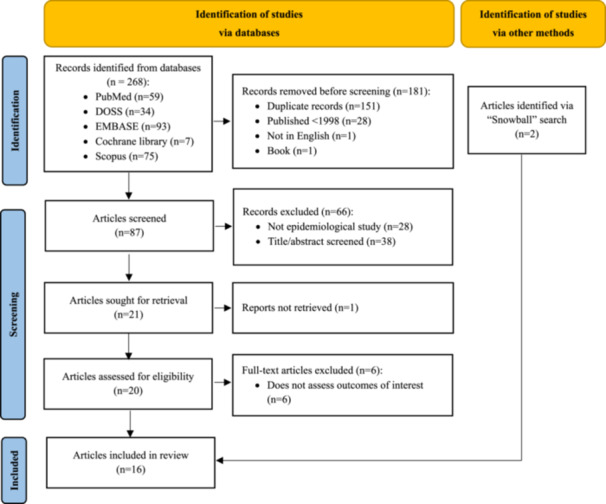
PRISMA flow diagram.

Overall, 268 articles were identified using the search strategy. Following the removal of duplicates and the discarding of articles based on the title and abstract screening, then the full‐text review where six articles were excluded (Udayamalee et al. 2024; Shimizu et al. 2023; Liew et al. 2020; Kerr et al. 2008; Carson et al. 1999; Hill et al. 1998) because they did not assess outcomes of interest (Supporting Information S2), 16 articles were ultimately included in the review (Figure 1).

### 3.1 Study Characteristics

The characteristics of the 16 studies are presented in Table 1. Nine of the 16 studies were cross‐sectional studies, five studies were retrospective, one was a prospective cohort study, and one was an ecological study. Many studies (10/16) were conducted over a 1‐year period. The majority of studies (12/16) were conducted in countries representing major world rugby nations: New Zealand (Welch et al. 2010; Marshall et al. 2005; Quarrie et al. 2005, 2020), Australia (Jagger et al. 2010; Ilia et al. 2014; Kroon et al. 2016), Japan (Tanaka et al. 2014; Nonoyama et al. 2020; Yamada et al. 1998), England (Jagger et al. 2010), and France (Muller‐Bolla et al. 2003).

**Table 1:** Study characteristics.

Reference	Year/period	Place	Study design	Sample characteristics	Sample size	Data collection
Lafferty et al. (2021)	2009–2018	United States	Longitudinal retrospective	Patients admitted in emergence department for rugby‐related orofacial injuries Males (*n* = 415) and females (*n* = 92) Age > 19 years: 20–64 years (mean: 24.5)	507	Data from National Electronic Injury Surveillance System (NEISS)
Padilha et al. (2021)	2019	Brazil	Cross‐sectional	16 best rugby union clubs in Brazil Players using a mouthguard Age: 18–40 years	244	Online questionnaire
Quarrie et al. (2020)	January 2005–December 2017	New Zealand	Longitudinal retrospective	Rugby injury claims Males and females Age: 5–40 years	635,657 claims	Data from the Accident Compensation Corporation (ACC) and New Zealand Rugby
Ruslin et al. (2016)	January 2000–April 2014	Netherlands	Longitudinal retrospective	Patients admitted to surgical department for sport‐related maxillofacial fractures Males (*n* = 85) and females (*n* = 23) Age: 10–64 years (mean: 30.6)	108	Data from the Department of Oral and Maxillofacial Surgery, Vrije Universiteit University Medical Center, Amsterdam
Kroon et al. (2016)	2012 season	Japan	Cross‐sectional	Junior rugby league players Males < 15 years (mean: 11.2)	494	Questionnaire
Nonoyama et al. (2020)	1 April 2006–31 March 2007	Australia	Longitudinal retrospective	Junior high school (*n* = 523,563) and high school students (*n* = 485,539)	1,009,102	Injury statistics from the Japan Sport Council based on the medical insurance system
Abdullah et al. (2015)	2009–2010	Malaysia	Cross‐sectional	Rugby players from tournaments of the Malaysian Rugby Union Males ≥ 16 years (mean: 22.7)	456	Self‐administered questionnaire
Ilia et al. (2014)	July 2010–July 2011	Australia	Cross‐sectional	Amateur rugby union players Age: 18–51 years (mean: 24.1)	225	Self‐administered questionnaire
Tanaka et al. (2014)	1 year period	Japan	Cross‐sectional	Males High school rugby players (mean age: 17.0 years) using custom‐made mouthguards (*n* = 69) Medical student rugby players (mean age: 22.5 years) using mouthguards of their choice (*n* = 431)	495	Self‐administered questionnaire
Schildknecht et al. (2012)	2010/2011 season	Swiss	Cross‐sectional	19 Swiss rugby league clubs Age: 10–47 years (mean: 23.1)	517	Interviewed with standardized questionnaire
Jagger et al. (2010)	2010	England–Australia	Cross‐sectional	All members of the first and second XV rugby squads at three secondary schools Males Age: 17–18 years	178	Self‐administered questionnaire
Welch et al. (2010)	From 1999 to 2008	New Zealand	Longitudinal retrospective	New dental injury claims from 19 sport categories Males (61.4%) and females (38.7%)	275,130 claims	Data from the Accident Compensation Corporation (ACC)
Quarrie et al. (2005)	1993–2003	New Zealand	Ecological	Dental injury claims of rugby players Age ≥ 16 years	327 claims in 1993 560 claims in 2002 774 claims in 2003	Data from the Accident Compensation Corporation (ACC) and telephone interviews
Marshall et al. (2005)	1993 season	New Zealand	Prospective cohort	Rugby union players Males (*n* = 240) and females (*n* = 87)	304	Weekly telephone interviews and data from the Rugby Injury and Performance Project (RIPP)
Muller‐Bolla et al. (2003)	1999–2000	France	Cross‐sectional	Rugby players in elite 1 (*n* = 12), elite 2 (*n* = 11) and national 1 (*n* = 22) clubs Mean age: 26.4 years	1,140	Self‐administered questionnaire
Yamada et al. (1998)	1993 and 1994 seasons	Japan	Cross‐sectional	Rugby players from 87 high school teams Males Age: 16 or 17 years	1,192	Self‐administered questionnaire

Six studies included both men and women (Welch et al. 2010; Marshall et al. 2005; Quarrie et al. 2020; Lafferty et al. 2021; Ruslin et al. 2016; Schildknecht et al. 2012), four included only men (Jagger et al. 2010; Kroon et al. 2016; Yamada et al. 1998; Abdullah et al. 2015), and the others did not specify the sex of the participants. Eleven studies (Welch et al. 2010; Marshall et al. 2005; Quarrie et al. 2020; Jagger et al. 2010; Kroon et al. 2016; Tanaka et al. 2014; Nonoyama et al. 2020; Yamada et al. 1998; Ruslin et al. 2016; Abdullah et al. 2015; Schildknecht et al. 2012) included junior participants (aged < 18 years).

Eight studies included players playing in clubs as members of a rugby team (Marshall et al. 2005; Jagger et al. 2010; Ilia et al. 2014; Kroon et al. 2016; Yamada et al. 1998; Muller‐Bolla et al. 2003; Padilha et al. 2021; Schildknecht et al. 2012), one study specified an amateur level of the participants (Ilia et al. 2014), and for the others, the level of play was not clear. The data from four studies did not exclusively target rugby but also other sports (Welch et al. 2010; Yamada et al. 1998; Lafferty et al. 2021; Ruslin et al. 2016); we only selected rugby‐related data from these studies.

### 3.2 Occurrence of Orofacial and Dental Injuries

The definitions of orofacial and dental injuries were unclear and not similar across the studies. The occurrences of orofacial and dental injuries are presented in Table 2.

**Table 2:** Occurrence of orofacial and dental injuries in rugby.

Reference	Orofacial injuries	Dental injuries
Lafferty et al. (2021)	507/507 patients (100%): inclusion criteria	4/507 orofacial injuries in rugby (0.8%)
	Injury location: – face and head: 456/507 (89.9%) – mouth: 36/507 (7.1%) – ear: 9/507 (1.8%) – eye: 5/507 (1.0%) – neck: 1/507 (0.2)	
Padilha et al. (2021)	84/244 rugby players (34.4%)	NR
Quarrie et al. (2020)	78,186/635,657 rugby injury claims (12.3%)	12,713/635,657 rugby injury claims (2.0%)
Ruslin et al. (2016)	9/108 patients with sport‐related maxillofacial fracture (8.3%)	NR
	Maxillofacial fractures by age: – 10–19 years: 0/9 (0%) – 20–29 years: 6/9 (67%) – 30–39 years: 1/9 (11%) – 40–49 years: 2/9 (22%) – ≥ 50 years: 0/9 (0%) Mandible fracture: 2/9 (22%)	
Nonoyama et al. (2020)	NR	24/3,795 boy students in high school playing rugby (0.6%)
Kroon et al. (2016)	266/494 rugby players (53.8%)	NR
Abdullah et al. (2015)	NR	Tooth fracture: 88/456 rugby players (19.3%) Tooth luxation: 30/456 rugby players (6.6%) Tooth avulsion: 5/456 rugby players (1.1%)
Ilia et al. (2014)	146/225 rugby players (64.9%), including 258 orofacial traumas: – lacerations to lips, cheeks and tongue: 115/258 (44.6%) – dental trauma: 108/258 (41.9%) – facial/mandibular fracture: 23/258 (8.9%) – dislocated jaw: 10/258 (3.9%)	108/258 rugby‐related orofacial trauma (41.9%): – crown or root fracture: 41/258 (15.9%) – luxation: 32/258 (12.4%) – bleeding socket: 20/258 (7.8%) – avulsion: 15/258 (5.8%) – Region commonly injured: – maxillary anterior teeth: 63.6% – mandibular anterior teeth: 19.2% – mandibular posterior teeth: 9.1% – maxillary posterior teeth: 8.1%
Tanaka et al. (2014)	NR	170/495 rugby players (34.3%): – 19/69 high school rugby players using custom‐made mouthguards (27.5%) – 151/426 medical student rugby players using mouthguards of their choice (35.4%)
Schildknecht et al. (2012)	204/517 rugby players (39.5%), including 249 facial injuries	35/517 rugby players (6.8%) – crown fracture: 26/517 – dislocations: 9/517 – avulsions: 3/517 Dental injuries by groups (*p* = 0.13): – national league (11.0%) – women's league (4.4%) Dental injuries by player positions (*p* = 0.048): – forwards (9.8%) – backs (4.7%) 193/517 had observed another player sustaining a dental injury during a game (37.3%)
	Facial injuries by groups (*p* < 0.001): – national league (58.7%) – premier league (43.4%) – women's league (30.0%) – junior league (22.0%) Facial injuries by player positions (*p* = 0.10): – forwards (45.1%) – backs (37.4%)	
Welch et al. (2010)	NR	75,110/275,130 new dental claims (27.3%) – 1999: 30.6% – 2000: 33.1% (highest) – 2001: 29.6% – 2002: 30.0% – 2003: 28.7% – 2004: 29.9% – 2005: 26.2% – 2006: 22.9% – 2007: 22.2% (lowest) – 2008: 24.4%
Jagger et al. (2010)	125/178 rugby players (70.2%)	46/178 rugby players (25.8%), including 62 dental injuries – fractured tooth: 20/178 (11.2%) – loosened tooth: 24/178 (13.5%) – avulsed tooth: 7/178 (3.9%)
	Head injury: – 17/178 (9.6%) unconscious – 19/178 (10.7%) conscious Facial injury: – 12/178 (6.7%) cut – 24/178 (13.5%) bruise – 9/178 (5.1%) fracture Lip injury: – 25/178 (14.0%) cut – 15/178 (8.4%) bruise	
Quarrie et al. (2005)	NR	1996: 2,6901997: 2,3161998: 2136/121,900 (1.8%)
Marshall et al. (2005)	Injuries to teeth, mouth or jaw: 6/304 rugby players (2.0%)	2/304 rugby players (0.7%)
Muller‐Bolla et al. (2003)	Trauma to the lower or middle face: 296/1140 rugby players (25.9%) – soft tissue: 7/1140 (0.6%) – teeth and periodontium: 206/1140 (18.1%) – lower or middle face fracture: 59/1140 (5.2%) – temporomandibular luxation: 24/1140 (2.1%)	206/1140 rugby players (18.1%) – fractures: 196/1140 (17.2%) – luxations: 10/1140 (0.9%) 6.8% of the trauma affected molars
Yamada et al. (1998)	673/1192 rugby players (56.5%) – intra‐oral laceration: 494/1192 (41.4%) – extra‐oral laceration: 356/1192 (29.9%) – fractured teeth: 100/1192 (8.4%) – luxation: 76/1192 (6.4%) – mandibular fracture: 6/1192 (0.5%) – mandibular dislocation: 5/1192 (0.4%) – other: 16/1192 (1.3%)	NR

Abbreviation: NR: not reported.

**Figure 2:**
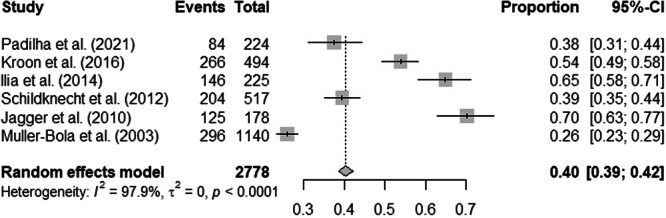
Meta‐analysis of the prevalence of orofacial injuries among rugby players.

Eleven studies reported the prevalence of orofacial injuries (Marshall et al. 2005; Quarrie et al. 2020; Jagger et al. 2010; Ilia et al. 2014; Kroon et al. 2016; Yamada et al. 1998; Muller‐Bolla et al. 2003; Lafferty et al. 2021; Padilha et al. 2021; Ruslin et al. 2016; Schildknecht et al. 2012). The overall prevalence of orofacial trauma was distributed as follows: one study had a prevalence rate of less than 30% (Muller‐Bolla et al. 2003), four studies had a prevalence rate between 30% and 60% (Kroon et al. 2016; Yamada et al. 1998; Padilha et al. 2021; Schildknecht et al. 2012), and two studies had a prevalence rate greater than 60% (Jagger et al. 2010; Ilia et al. 2014). Only studies that collected data from club rugby players by questionnaires (Jagger et al. 2010; Ilia et al. 2014; Kroon et al. 2016; Muller‐Bolla et al. 2003; Padilha et al. 2021; Schildknecht et al. 2012) were included in the meta‐analysis to calculate the overall prevalence of orofacial injuries which was 40.4% (95% CI: 38.5–42.2) (Figure 2).

Among orofacial traumas, the prevalence of injuries to soft tissues (lacerations, lips, tongue, cheeks, and teeth) was presented in seven studies (Marshall et al. 2005; Jagger et al. 2010; Ilia et al. 2014; Yamada et al. 1998; Muller‐Bolla et al. 2003; Lafferty et al. 2021; Schildknecht et al. 2012). Mandibular damage was presented in six studies (Marshall et al. 2005; Ilia et al. 2014; Yamada et al. 1998; Muller‐Bolla et al. 2003; Lafferty et al. 2021; Ruslin et al. 2016), head and face injuries were reported in six studies (Quarrie et al. 2020; Jagger et al. 2010; Ilia et al. 2014; Muller‐Bolla et al. 2003; Ruslin et al. 2016; Schildknecht et al. 2012), and two studies simply reported the prevalence of orofacial lesions without specifying the location (Kroon et al. 2016; Padilha et al. 2021). Only one study presented results based on players’ gaming experience. Padilha et al. reported that since players started playing rugby, the prevalence of orofacial trauma was 25.5% for players who played for less than 1 year, 26.8% for players who played for 1–2 years, 38.6% for players who played for 2–4 years, and 41.2% for players who played for more than 4 years (Padilha et al. 2021).

Two studies reported the rate of claims due to orofacial injuries. Quarrie et al. (2005) reported rates of orofacial injuries of 0.7/1000 player‐hours during matches and 0.1/1000 player‐hours during practices. Quarrie et al. (2020) reported that the claim rate for orofacial injuries was 347/1000 players/year (208/1000 female players/year and 363/1000 male players/year), including a claim rate for dental injuries of 6.8/1000 players/year.

**Figure 3:**
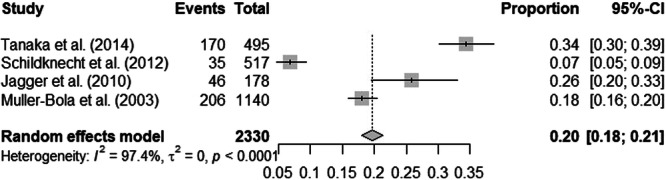
Meta‐analysis of the prevalence of dental injuries among rugby players.

Four studies asked club rugby players via questionnaires regarding their prevalence of dental injuries (Jagger et al. 2010; Tanaka et al. 2014; Muller‐Bolla et al. 2003; Schildknecht et al. 2012), and two studies presented the percentage of claims linked to rugby‐related injuries (Welch et al. 2010; Quarrie et al. 2020). Only studies that collected data from club rugby players by questionnaires (Jagger et al. 2010; Tanaka et al. 2014; Muller‐Bolla et al. 2003; Schildknecht et al. 2012) were included in the meta‐analysis to calculate the overall prevalence of orofacial injuries which was 19.6% (95% CI: 18.1–21.3) (Figure 3).

Five studies specified the type of dental injury: four studies showed that among dental injuries, fractured teeth were the most common injury (Ilia et al. 2014; Muller‐Bolla et al. 2003; Abdullah et al. 2015; Schildknecht et al. 2012), while in the study of Jagger et al. (2010) the most common dental injury was tooth loss (13.5% of dental injuries). One study mentioned that the dental region most affected during injuries was the maxillary anterior teeth (63.6%) (Ilia et al. 2014).

In the study of Schildknecht et al. (2012) national league players experienced a greater percentage of dental injury (11.0%) than women's league players (4.4%). Regarding claims to the insurance, 38.7% were made by females, and 61.3% were made by males (Welch et al. 2010). This can be explained by the greater number of men playing rugby. Indeed, Quarrie et al. (2020) showed that 10% of players were female, and they sustained 6% of the injuries. In general, male players had higher claim rates than female players regarding general injuries (not exclusively dental injuries).

Only one study provided information on the occurrence of dental injuries depending on the player's playing position: there was a significant difference in dental injuries sustained by forwards (9.8%) compared to backs (4.7%) (*p* < 0.05) (Schildknecht et al. 2012).

According to Ilia et al. (2014) complications following dental trauma occurred in 24.7% of patients. Complications included the following: the tooth was lost/extracted (38.1%), the tooth changed color (28.6%), additional treatment was required (23.8%), and infections occurred (9.5%).

The certainty in cumulative evidence assessed by GRADE was considered very low for orofacial and dental injuries (Table 3). The “risk of bias” topic was categorized as “serious” because of concerns regarding outcome measures, and the “inconsistency” topic was categorized as “very serious” due to the high heterogeneity of the meta‐analysis.

**Table 3:** Grading of recommendations assessment, development and evaluation (GRADE). Evidence profile.

Certainty assessment							Summary of findings		
No of studies	Study design	Risk of bias	Inconsistency	Indirectness	Imprecision	Other considerations	Sample	Pooled prevalence [95% CI]	Certainty of evidence
Prevalence of orofacial injuries									
6	Observational studies	Serious	Very serious	Not serious	Not serious	None	2778	40.4% [38.5–42.2]	⊕**◯◯◯** Very low
Prevalence of dental injuries									
4	Observational studies	Serious	Very serious	Not serious	Not serious	None	2,330	19.6% [18.1–21.3]	⊕**◯◯◯** Very low

Abbreviation: CI: confidence interval.

### 3.3 Use of Mouthguards

The use of mouthguards is presented in Table 4. Ten studies reported the prevalence of wearing mouthguards (Marshall et al. 2005; Quarrie et al. 2005; Jagger et al. 2010; Ilia et al. 2014; Kroon et al. 2016; Tanaka et al. 2014; Yamada et al. 1998; Muller‐Bolla et al. 2003; Padilha et al. 2021; Schildknecht et al. 2012). In two studies (Jagger et al. 2010; Padilha et al. 2021), the rate of use was 100%, but in one of these studies, the use of a mouthguard was an inclusion criterion (Padilha et al. 2021). Seven studies mentioned the wearing percentages of different types of mouthguards: four indicated that the “boil and bite” type was the most commonly used (Ilia et al. 2014; Kroon et al. 2016; Yamada et al. 1998; Padilha et al. 2021), while the other three reported that the most commonly used type was “custom‐made” (Tanaka et al. 2014; Muller‐Bolla et al. 2003; Schildknecht et al. 2012). Five studies described when mouthguards were worn: the most frequent was during competition only, followed by during competition and training, and the least frequent was during training only (Quarrie et al. 2005; Ilia et al. 2014; Kroon et al. 2016; Yamada et al. 1998; Muller‐Bolla et al. 2003).

**Table 4:** Use of mouthguards among rugby players.

Reference	Percentage of mouthguard use	Relative rate and risk	Mouthguard form	Time to wear mouthguard
Padilha et al. (2021)	244/244 rugby players (100%): inclusion criteria	NR	Boil and bite: 198/244 (81.2%) Ready to wear: 24/244 (9.8%) Custom‐made: 22/244 (9.0%)	NR
Kroon et al. (2016)	337/494 rugby players (68.2%) – under 8: 54.5% – under 9: 64.6% – under 10: 71.9% – under 11: 73.2% – under 12: 74.8% – under 13: 68.8% – under 14: 46.4% – under 15: 73.9%	NR	Boil and bite: 218 (64.7%) Custom‐made: 78 (23.1%) Ready to wear: 41 (12.2%)	Training only: 4/494 Games only: 201/494 Training and games: 87/494 When told by parents/coaches: 13/494 Never: 28/494
Ilia et al. (2014)	133/225 rugby players (76.9%)	75.5% and 24.5% of players experiencing injuries in competition and training, respectively	Boil and bite: 58.4% Custom‐made: 41.0% Stock: 0.6%	Competition: 72/173 (57.2%) Competition and training: 99/173 (41.6%) Training only: 2/173 (1.2%)
Tanaka et al. (2014)	High school rugby players: 52% Medical students rugby players: 34%	Frequency of mouthguard use associated with incidence of oral injury:	High school rugby players: 100% custom‐made (inclusion criteria) Medical student rugby players: 85.2% custom‐made	Training
		High school rugby players: – < 44%: OR = 1 – 44%–50%: OR = 0.49 – 50%–66%: OR = 0.19 – > 66%: OR = 0.11 Medical student rugby players: – < 5%: OR = 1 – 5%–50%: OR = 0.69 – 50%–82%: OR = 0.53 – > 82%: OR = 0.27		
Schildknecht et al. (2012)	88.2% of rugby players – female players: 94.4% – juniors: 84.0%	NR	Custom‐made: 76.5% Stock: 23.5%	NR
Jagger et al. (2010)	100% of rugby players	NR	NR	NR
Quarrie et al. (2005)	1993: 67% 2002: 85% 2003: 93%	Relative risk of claims for wearers vs. nonwearers: 4.6 (CI: 3.8–5.6) Relative risk of injury for wearers vs. nonwearers among junior players: 2.7 (CI: 1.6–4.6)	NR	2002: 85% in games and 38% in practices 2003: 93% in games and 59% in practices
Marshall et al. (2005)	65% of rugby players	Rate ratio adjusted for orofacial injury (teeth, mouth and jaw) by mouthguard usage: 0.56 (CI: 0.07–4.63)	NR	NR
Muller‐Bolla et al. (2003)	733/1140 rugby players (64.3%)	NR	Custom‐made: 421/733 (57.4%) Mouth formed: 312/733 (42.6%)	Systematically: 496/733 During competition: 213/733 One from time to time: 24/733
Yamada et al. (1998)	269/1192 (22.6%)	NR	Stock: 2.1% Mouth‐formed: 89.3% Custom‐made: 8.6%	Games and practices: 5.2% Games only: 17.8% Practices only: 1.7% Sometimes: 11.5% Not using: 63.6%

Abbreviations: CI: confidence interval; NR: not reported; OR: odds ratio.

### 3.4 Management of Tooth Injury

Four studies focused on the management of tooth injury after incidents (Ilia et al. 2014; Kroon et al. 2016; Abdullah et al. 2015; Schildknecht et al. 2012). In the study of Ilia et al. (2014), 51.4% of injured players did not receive treatment, 19.2% consulted a dentist, and 17.8% consulted a doctor and a dentist. In the study of Abdullah et al. (2015) immediately seeing a dentist was the most common response for the management of tooth fracture (47.8%) and tooth luxation (48.7%). For an avulsed tooth, 35.1% did not look for the tooth and did not see the dentist unless there was pain, 33.1% did not look for the tooth but saw the dentist immediately, and 23.2% kept the tooth and brought it to see the dentist immediately. According to a study by Schildknecht et al. (2012), 69.6% of rugby players would visit a dentist with an avulsed tooth, while others (30.4%) would visit a hospital; 60.5% were aware that an avulsed tooth can be replanted. Kroon et al. (2016) collected information about the management of an avulsed tooth: for 50.4% of young rugby players, only a dentist can put it back, and most of them (48.6%) kept the tooth in water, while for 40.6% of the coaches, 15 min is the time limit to put back an avulsed tooth (minimum time limit proposed by the questionnaire), and most of them (59.4%) kept the tooth in milk.

### 3.5 Risk of Bias

The details of the critical appraisal of all studies are presented in Table 5. With regard to the risk of bias in the individual studies, most (11/16) were judged as moderate risk (Welch et al. 2010; Marshall et al. 2005; Quarrie et al. 2005, 2020; Jagger et al. 2010; Ilia et al. 2014; Kroon et al. 2016; Yamada et al. 1998; Padilha et al. 2021; Abdullah et al. 2015; Schildknecht et al. 2012), five studies as low risk (Tanaka et al. 2014; Nonoyama et al. 2020; Muller‐Bolla et al. 2003; Lafferty et al. 2021; Ruslin et al. 2016), and none as high risk.

**Table 5:** Risk of bias.

Reference	Q1	Q2	Q3	Q4	Q5	Q6	Q7	Q8	Score yes (%)	Risk of bias
Lafferty et al. (2021)	YES	YES	YES	YES	NO	NO	YES	YES	75.0	Low
Padilha et al. (2021)	YES	YES	NO	YES	NO	NO	NO	YES	50.0	Moderate
Quarrie et al. (2020)	YES	YES	NO	YES	NO	NO	NO	YES	50.0	Moderate
Ruslin et al. (2016)	YES	YES	YES	YES	NO	NO	YES	YES	75.0	Low
Kroon et al. (2016)	YES	YES	YES	YES	NO	NO	NO	YES	62.5	Moderate
Nonoyama et al. (2020)	YES	YES	YES	YES	NO	NO	YES	YES	75.0	Low
Abdullah et al. (2015)	YES	YES	YES	YES	NO	NO	NO	YES	62.5	Moderate
Ilia et al. (2014)	YES	YES	YES	YES	NO	NO	NO	YES	62.5	Moderate
Tanaka et al. (2014)	YES	YES	YES	YES	YES	YES	NO	YES	87.5	Low
Schildknecht et al. (2012)	YES	YES	YES	YES	NO	NO	NO	YES	62.5	Moderate
Jagger et al. (2010)	YES	NO	YES	YES	NO	NO	NO	YES	50.0	Moderate
Welch et al. (2010)	YES	YES	YES	YES	NO	NO	NO	YES	62.5	Moderate
Quarrie et al. (2005)	YES	YES	YES	YES	NO	NO	NO	YES	62.5	Moderate
Marshall et al. (2005)	YES	YES	YES	YES	NO	NO	NO	YES	62.5	Moderate
Muller‐Bolla et al. (2003)	YES	YES	YES	YES	YES	YES	NO	YES	87.5	Low
Yamada et al. (1998)	YES	YES	YES	YES	NO	NO	NO	YES	62.5	Moderate

*Note:*

Q1: Were the criteria for inclusion in the sample clearly defined?

Q2: Were the study subjects and the setting described in detail?

Q3: Was the exposure measured in a valid and reliable way?

Q4: Were objective, standard criteria used for measurement of the condition?

Q5: Were confounding factors identified?

Q6: Were strategies to deal with confounding factors stated?

Q7: Were the outcomes measured in a valid and reliable way?

Q8: Was appropriate statistical analysis used?

## 4 Discussion

Rugby has been played worldwide for decades. However, the scientific evidence presented in this review mainly comes from three countries that have played this sport for many years, namely, New Zealand, Australia, and Japan (Welch et al. 2010; Marshall et al. 2005; Quarrie et al. 2005, 2020; Jagger et al. 2010; Ilia et al. 2014; Kroon et al. 2016; Tanaka et al. 2014; Nonoyama et al. 2020; Yamada et al. 1998). Only seven studies included rugby players from other countries (Jagger et al. 2010; Muller‐Bolla et al. 2003; Lafferty et al. 2021; Padilha et al. 2021; Ruslin et al. 2016; Abdullah et al. 2015; Schildknecht et al. 2012), which is surprising for countries as important in the field of rugby as France or England, where we could expect more interest in the field.

Given the potential nature of rugby injuries, a strong understanding of those injuries is important for successful injury risk minimization and prevention strategies. Yeomans et al. (2018) reported that the overall incidence of match injuries among senior amateur rugby union players was 46.8/1000 player‐hours. In the literature, many studies have focused on injuries to different body parts in rugby, but few have focused on the orofacial sphere. However, Arif et al. (2023) reported that the head and face were the most common sites of rugby‐related injury in both sexes.

The occurrence of orofacial trauma in rugby players is frequent because, in the meta‐analysis, we found that the overall prevalence of orofacial injuries was 40.4%. Higher prevalences were reported in the studies of Jagger et al. (2010) and Ilia et al. (2014) with 70.2% and 64.9%, respectively. However, these two studies focused on young rugby players, and it is possible that age influences how they are attentive to and measured in games. The rate of facial trauma seems to be correlated with the number of years of play (Padilha et al. 2021).

The occurrence of dental trauma in rugby players is also frequent because, in the meta‐analysis, we found that the overall prevalence of dental injuries was 19.6%. Among dental injuries, tooth fracture was the most common injury (Ilia et al. 2014; Muller‐Bolla et al. 2003; Abdullah et al. 2015; Schildknecht et al. 2012), which could be explained by the physical nature of the shocks perceived by the players. Bird et al. (1998) and Garraway et al. (2000) suggested that the tackle phase of play accounted for the majority of injuries. The impacts of such injuries on the physical and mental health of a player are an essential point of consideration. However, there are no studies on a player's downtime after a dental injury or on the impact of a dental injury on the game.

In comparison with other sports, rugby appears as a sport with a high prevalence of orofacial and dental injuries. In the meta‐analysis of Werlich et al. the prevalence of dentofacial and dental injuries in contact sports players were 27.6% (95% CI: 17.9–38.5) and 19.6% (95% CI: 8.1–34.6), respectively. The prevalences dentofacial injuries were 37.4% (95% CI: 17.5–59.8) in rugby, 27.3% (95% CI: 9.5–50.1) in basketball, 24.6% (95% CI: 14.9–35.8) in handball, and 19.1% (95% CI: 6.8–35.6) in field hockey (Oliveira Werlich et al. 2020). In the meta‐analysis of Polmann et al. the prevalence of dentofacial and dental injuries in combat sports practitioners was 30.3% (95% CI: 18.1–44.1) and 25.2% (95% CI: 12.3–40.8), respectively. The prevalences dentofacial injuries were 52.9% (95% CI: 37.9–67.8) in jiu‐jitsu, 45.9% (95% CI: 25.4–67.1) in boxing, 43.5% (95% CI: 30.0–57.5) in karate, 37.5% (95% CI: 20.8–56.0) in Taekwondo, and 25.0% (95% CI: 7.6–48.2) in Judo (Polmann et al. 2020). In the meta‐analysis of de Oliveira et al. the prevalence of orofacial and dental injuries in wheeled non‐motor sport athletes was 21.7% (95% CI: 8.7–34.7) and 7.5% (95% CI‐4.3; 10.7), respectively (de Oliveira et al. 2021).

Epidemiological data on rugby injuries are mainly limited to individuals at the elite level, adults and men. There are few data on women and children. This may be due to the prevalence of male athletes in rugby. However, according to World Rugby, the share of these populations tends to increase over time (world. rugby 2025). It is important for future studies to consider all age groups, all levels of play and female cohorts to gain more knowledge and understanding of the similarities and differences in rugby injury epidemiology. The increased size and strength of male players may result in greater forces in contact events than those of youth, adolescent or female players, which would probably explain the different orofacial injury rate data of these populations (Bartolomei et al. 2021; Baker 2002).

In this review, the varying findings regarding the prevalence and types of mouthguards use in rugby highlighted the complexity of the factors influencing players’ adherence to protective measures. A higher rate was reported in the Swiss study of Schildknecht et al. (2012) with a voluntary wearing rate of 88.2%. The use of mouthguards increased by 26% from 1993 (67%) to 2003 (93% in games and 59% in training) in New Zealand (Quarrie et al. 2005), showing better use since 1998 when it became compulsory for rugby players to wear mouthguards during matches. This rule likely contributes to disparities in the use of mouthguards during training and matches. Indeed, the prevalence of wearing a mouthguard is higher during matches than during training (Ilia et al. 2014; Kroon et al. 2016; Muller‐Bolla et al. 2003). The use of mouthguards is crucial because Quarrie et al. (2005) revealed a greater relative rate of injury among junior players who did not use mouthguards. Kroon et al. (2016) reported that coaches should encourage the use of mouthguards for juniors, suggesting a potential gap between awareness and actual compliance among young players.

Regarding the mouthguard type, rugby players reported different preferences. This gap may be attributed to personal preferences, comfort, cost, or accessibility. A 2017 study developed a scale of protection for different types of mouthguards and found that custom‐made mouthguards were among the most effective for sports‐related trauma prevention (Green 2017). This type also allows an increase in the performance of athletes (Cao et al. 2023). Custom‐made was the most commonly used type of mouthguard in only three studies included in this review (Tanaka et al. 2014; Muller‐Bolla et al. 2003; Schildknecht et al. 2012).

The recent emergence of “smart mouthguards” in the rugby world offers fertile ground for in‐depth investigations. These innovative devices, which integrate technologies such as shock sensors and real‐time monitoring systems and were initially designed to measure head accelerations and are placed at the center of the forces experienced by each player, could also be promising proactive approaches for preventing oral injuries (Bridgman et al. 2019; Liu et al. 2020). Future studies could therefore contribute to reviewing and optimizing oral protection strategies in rugby. By combining the benefits of modern technology with traditional prevention, this research could pave the way for significant advances in reducing dental trauma and improving the overall safety of rugby players.

The strength of this systematic review is that it contributes to the current level of knowledge regarding the prevalence and prevention of rugby‑related orofacial and dental trauma. However, this review is not without limitations. A notable constraint is the heterogeneity of the included studies in terms of methodologies, sample sizes, different proportions of mouthguard use, and the focus on certain demographic groups, such as age, sex and location. Moreover, the heterogeneity was high in the meta‐analysis. This diversity may introduce variability in reported prevalence rates and limit the generalizability of the results. In addition, the use of self‐assessment in numerous studies introduces a risk of bias because participants may underestimate the number of orofacial injuries suffered, particularly the less serious and less painful injuries, and also overestimate their adherence to the use of mouthguards. Additionally, variations in the definitions of mouthguard use and injury types between studies may hamper direct comparisons. These limitations hinder the ability to draw comprehensive conclusions about the entire rugby playing population and conducted to a very low level of certainty regarding GRADE.

## 5 Conclusion

This study presents a comprehensive review of the literature reporting orofacial and dental injuries in rugby since 1998. The prevalence of orofacial injuries varies among different studies but is rather high in general. The most common orofacial injuries involve the soft tissues, while a fractured tooth is the most commonly reported dental injury. This review also highlights that rugby players do not wear enough mouthguards and that they have little knowledge of how to manage dental injuries during sporting activities. Information on the use of mouthguards and the management of dental injuries is needed among rugby players.

## Author Contributions

Conceptualization: Alexandre Baudet and Marc Chaboussie. Methodology: Alexandre Baudet. Formal analysis: Alexandre Baudet and Marc Chaboussie. Investigation: Alexandre Baudet and Marc Chaboussie. Data curation: Alexandre Baudet and Marc Chaboussie. Writing—original draft preparation: Alexandre Baudet and Marc Chaboussie. Writing—review and editing: Alexandre Baudet and Marc Chaboussie. Supervision: Alexandre Baudet. Project administration: Alexandre Baudet.

## Funding

The authors received no specific funding for this work.

## Ethics Statement

The authors have nothing to report.

## Consent

The authors have nothing to report.

## Conflicts of Interest

The authors declare no conflicts of interest.

## Supporting information


**Supplementary data S1:** Research questions and selection criteria. **Supplementary data S2:** Reasons for exclusion during full‐text review.

## Data Availability

Data sharing is not applicable to this article as no new data were created in this study.
