# Supplementary material for: Occurrence of Orofacial and Dental Injuries in Rugby: Systematic Review and Meta‐Analysis
Source: Clin Exp Dent Res. 2026 Mar 23;12(2):e70315. doi: 10.1002/cre2.70315 (PMC13140634; doi:10.1002/cre2.70315)
Supplement: Supplementary file 1 — Supplementary data S1: Research questions and selection criteria. Supplementary data S2: Reasons for exclusion during full‐text review. [file CRE2-12-e70315-s001.docx]

**Occurrence of orofacial and dental injuries in rugby:
Systematic review and meta-analysis**

# Supplementary data S1: Research questions and selection criteria

## Research questions

Main research question:

- What is the occurrence (prevalence/incidence) of orofacial and dental injuries in rugby?
- PICO terms (Population, Intervention, Comparison, Outcome):
  - P: Rugby players of all genders of any age and all playing levels
  - I: Not applicable
  - C: Not applicable
  - O: Occurrence (prevalence/incidence) of orofacial and dental injuries in rugby

Second research questions:

- Among articles reporting orofacial or dental injuries in rugby, what is prevalence of mouthguard use?
- Among articles reporting orofacial or dental injuries in rugby, what is the knowledge of rugby players to manage a tooth injury?

## Selection criteria

| Inclusion criteria | Exclusion criteria |
| --- | --- |
| - Articles published from 1998 to 31^st^ March 2025 - Observational epidemiological studies (retrospective, longitudinal, cohort, ecological and cross-sectional studies) - Articles in English-language - Articles on orofacial trauma and/or dental injuries during rugby - Articles reporting the prevalence or incidence of orofacial or dental injuries among rugby players - Articles including individuals of all genders of any age and all playing levels | - Books, review articles, qualitative studies, case reports and case series |

# Supplementary data S2: Reasons for exclusion during full-text review

| Auteurs (year). Title | Reason for exclusion |
| --- | --- |
| Udayamalee et al. (2024). Oro-dental trauma burden and mouthguard usage among contact sports players: A call for sports dentistry initiatives in Sri Lanka | Study reported injuries from different sports (hockey, football, rugby, karate, taekwondo and boxing) without specific description of injuries among rugby players |
| Shimizu et al. (2023). Quantitative text analysis of the mechanisms of tooth injury: Analysis of accidents in five sports that occurred in 15 years under school control | Study included injuries cases for five ball sports (baseball, soccer, basketball, volleyball, and rugby). It did not report the prevalence or incidence of orofacial or dental injuries among rugby players |
| Liew et al. (2020). Effectiveness of an educational workshop in improving knowledge on dental trauma among rugby players | Study focused on emergency training of rugby players. It did not report orofacial trauma and/or dental injuries during rugby |
| Kerr et al. (2008). Collegiate rugby union injury patterns in New England: a prospective cohort study | Study reported incidence of injuries from different body sites among men and women. It did not report the incidence of orofacial or dental injuries among all rugby players. |
| Carson et al. (1999). The epidemiology of women’s rugby injuries | Study reported injuries from different body sites (head and neck, upper extremity, shoulders, trunk and lower extremity) without specific description of orofacial trauma |
| Hill et al. (1998). A one-year review of maxillofacial sports injuries treated at an accident and emergency department | Study reported injuries from different sports (rugby, cycling, football, ice sports, raquet sports, cricket, hockey, contact sports, golf and other) without specific description of injuries among rugby players |
